# Coverage and factors associated with receipt of early newborn postnatal care components in Afghanistan: findings from the 2022–2023 multiple indicator cluster survey

**DOI:** 10.1080/16549716.2026.2710553

**Published:** 2026-08-03

**Authors:** Essa Tawfiq, Muhammad Haroon Stanikzai, Zainab Ezadi, Massoma Jafari, Fateme Dadras, Omid Dadras

**Affiliations:** a The Kirby Institute, UNSW Sydney, Sydney, Australia; b Department of Public Health, Faculty of Medicine, Kandahar University, Kandahar, Afghanistan; c Master of Science in Midwifery, Reproductive Health, Kabul, Afghanistan; d Health Profession Education Research, University of Toronto, Toronto, Ontario, Canada; e Department of Obstetrics and Gynecology, Faculty of Medicine, Alborz University of Medical Sciences, Karaj, Iran; f Research Centre for Child Psychiatry, University of Turku, Turku, Finland

**Keywords:** Early newborn care, determinants, composite outcome, Afghanistan, newborn postnatal care

## Abstract

**Background:**

Neonatal mortality remains high in low‑ and middle‑income countries, where early postnatal care (PNC) within 48 hours can avert many deaths. Yet neither the national coverage of newborn PNC nor the factors associated with its receipt have been assessed.

**Objectives:**

We assessed the coverage of newborn PNC components and the factors associated with their receipt in Afghanistan.

**Methods:**

We conducted a cross‑sectional analysis of 11,965 mother–infant dyads from the 2022–2023 Afghanistan Multiple Indicator Cluster Survey. The outcome was coded ‘yes’ if the infant received at least two of the following six newborn PNC components within 2 days of birth: temperature measurement, weighing, cord examination, breastfeeding counseling, observation of breastfeeding, and counseling on danger signs. We fitted a multivariable logistic regression model to identify factors associated with the outcome.

**Results:**

Only 41.0% of infants received ≥2 components; merely 1.9% received all six components of PNC. The odds of receiving ≥2 components of PNC were higher among infants whose households had an educated head, whose mothers attended 1–3 or ≥4 antenatal care (ANC) visits, those born in private and public health facilities, and those delivered by cesarean section. Infants in higher wealth quintiles also had progressively greater odds. Conversely, infants perceived as small at birth had lower odds of receiving ≥2 PNC components.

**Conclusion:**

Four out of ten newborns received ≥2 components of PNC, with marked inequities by head of household education, ANC use, delivery setting, and wealth status. Targeted interventions addressing these inequalities are urgently needed to strengthen early newborn PNC.

## Paper Context

**Main findings:** Four out of ten newborns in Afghanistan received at least two components of early postnatal care, with substantial inequalities by head of household education, antenatal care use, place of birth, birth size, and household wealth.

**Added knowledge:** This study provides nationally representative evidence on the receipt and associated factors of early newborn postnatal care in Afghanistan, moving beyond previous work that mainly examined individual newborn care components or maternal postnatal care.

**Global health impact for policy and action:** Strengthening antenatal care, facility-based delivery, postnatal care quality, and targeted support for small and disadvantaged newborns can help improve newborn survival and reduce inequities in fragile health systems.

## Background

The immediate postnatal period, particularly the first 48 hours, is crucial for both mothers and newborns due to the numerous physical and emotional changes that necessitate careful attention [1]. According to the World Health Organization (WHO), postnatal care (PNC) services for newborns should start as soon as possible after birth as many neonatal deaths occur within the first 48 hours of life [2], and most of these deaths can be prevented by providing quality early newborn PNC services [3,4]. However, global coverage of early newborn PNC services within this timeframe is only 64%, with considerable variations between countries [5]. Additionally, several studies have reported that the quality of early newborn PNC services is at suboptimal levels in many low- and middle-income countries (LMICs) [6–9].

Globally, about 2.8 million newborns die during the neonatal period each year. Of these deaths, approximately 75% occur within the first week of life and are classified as early neonatal deaths, while around 1 million neonatal deaths occur within the first 24 hours after birth [10–12]. In 2021, Afghanistan had one of the world’s highest newborn mortality rates, at 37 deaths per 1000 live births [13], reflecting substantial gaps in the coverage and quality of neonatal and child healthcare services [14]. Afghanistan is committed to achieving the Sustainable Development Goal (SDG) target for neonatal mortality, aiming to reduce it to 12 deaths per 1,000 live births by 2030. To reach this goal, the country must substantially improve newborn care, particularly in enhancing the quality of early newborn PNC services [15].

Timely and efficient newborn care services can significantly reduce neonatal morbidity and mortality in LMICs [16]. The updated 2022 WHO guidelines emphasize the importance of early newborn PNC services (within the first two days after birth) as a crucial component of maternal and child healthcare services [5]. Early newborn PNC services include measuring the infant’s temperature, providing cord care, counseling on breastfeeding, observing breastfeeding, monitoring of growth and development, counseling on danger signs, and screening for congenital diseases [17,18]. Numerous studies demonstrate that the quality of early newborn PNC services significantly impacts neonatal health and survival [16,19,20].

Despite the well-established benefits, the uptake of early newborn PNC services remains suboptimal in many LMICs. For instance, a recent study in Ethiopia found that only 39% of infants received two components of early newborn PNC services in the first 2 days after birth [21]. Similarly, studies have shown that the uptake of early newborn PNC services is low in many LMICs, ranging from 9% to 63% [22–24]. Moreover, several factors are associated with the uptake of early newborn PNC services, including maternal education, residential area, antenatal care (ANC) utilization, mode and place of delivery, baby size and weight, media exposure, and household wealth status [1,21,25,26].

Although previous research in Afghanistan has examined the proportion of newborns receiving essential newborn care [19,27–30], few in-depth studies have assessed the receipt of early newborn PNC components. To address this gap, the present study examined the coverage of these components and the factors associated with their receipt using secondary data from the Afghanistan Multiple Indicator Cluster Survey (MICS) 2022–2023.

## Methods

### Study design and data source

**Figure 1.**
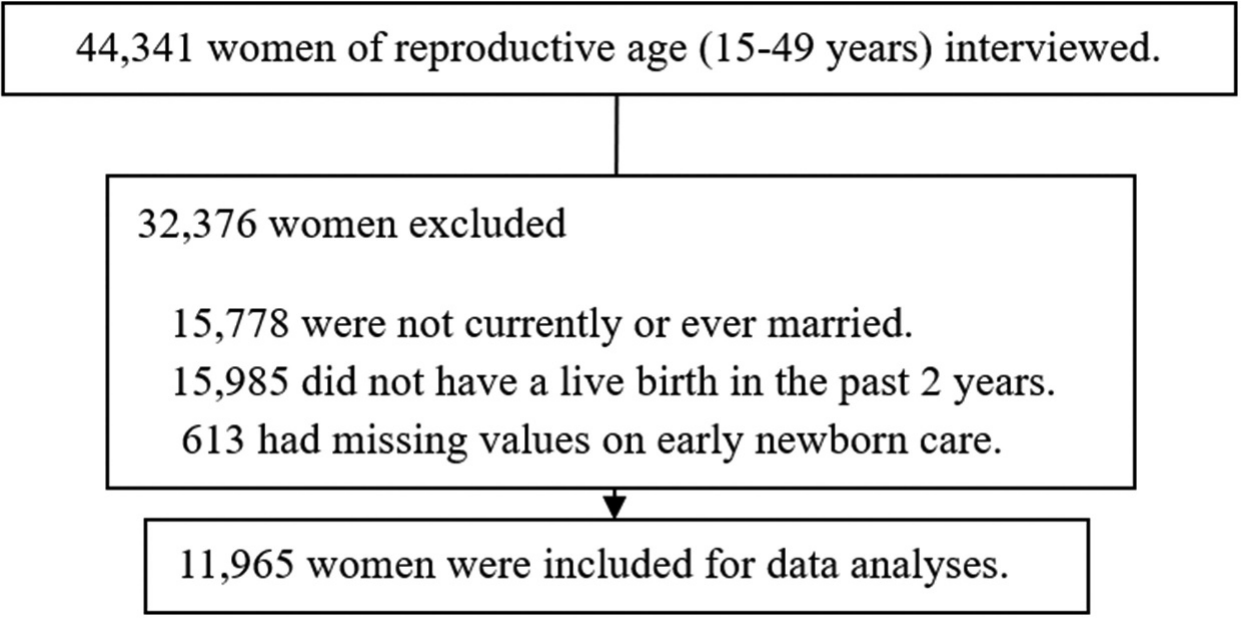
Flow diagram of sample selection.

In this cross-sectional study, we used data from the Afghanistan MICS 2022–2023, accessed on 10 May 2025 [31]. The MICS 2022–2023 used a two-stage cluster sampling approach and collected data from a nationally representative sample. The survey design, sampling method, and data collection are reported as part of the MICS 2022–2023 report [31]. During the MICS survey, trained surveyors collected data from women of reproductive age (15–49 years), who answered questions related to child and maternal health and nutrition. For this study, we included data from 11,965 ever-married women of reproductive age who had a live birth over the past 2 years prior to the MICS 2022–2023 survey (Figure 1).

## Study variables

### Outcome variable

The primary outcome was the receipt of early newborn PNC components and was defined as whether the newborn received at least two of the following six recommended components within the first 2 days after birth [2,21]: (i) measuring the newborn’s temperature, (ii) weighing the newborn, (iii) examining the umbilical cord, (iv) advising mothers on breastfeeding, (v) observing mothers while breastfeeding, and (vi) advising mothers on newborn danger signs. The binary outcome variable, ≥2 components vs. 0–1 component, was used in the regression analysis.

### Explanatory variables

The explanatory variables examined were women’s age at the time of the survey (15–29, 30–39, and 40–49 years), women’s education level (no formal education, attended formal education), education of the household head (no formal education, attended formal education), household wealth index (from the lowest to the highest quintile), residential area (urban vs. rural), ANC use (no visit, 1–3 visits, ≥4 visits), infant sex (male vs. female), birth order (firstborn versus second- or higher-order birth), place of delivery (whether the woman gave birth at home, at a public health facility, or a private health facility), perceived small-size baby at birth (whether the mother perceived her newborn as small at birth), cesarean section for the most recent birth (yes vs. no), and media access was coded ‘yes’ if the woman watched television daily, listened to the radio daily, or read a newspaper; otherwise, it was coded ‘no’.

### Statistical analysis

We used descriptive statistics to summarize the sociodemographic characteristics of the women and their newborns. Chi-square tests were used to assess associations between the explanatory variables and receipt of early newborn PNC components. To examine the likelihood of receiving early newborn PNC services across the categories of explanatory variables, bivariate, and multivariable binary logistic regression models were specified and run. The selection of explanatory variables was based on the literature [21,23,25,32]. To determine the inclusion of explanatory variables in the multivariable analysis, we included in the multivariable regression analysis any explanatory variable with a *p*-value < 0.25 across all categories in the bivariate model. A *p*-value < 0.25 has been used to select explanatory variables for multivariable analysis in previous studies [33]. We assessed multicollinearity among explanatory variables by calculating variance inflation factor (VIF), and found no evidence of problematic multicollinearity. We accounted for the complex survey design and sampling weights by specifying the survey strata and primary sampling units. We provided crude as well as adjusted odds ratios and 95% confidence intervals (CI) from the bivariate and multivariable logistic regression analyses. As a post-hoc sensitivity analysis, we conducted two additional analyses using alternative cut-off points (≥4 vs. 0–3 components and ≥3 vs. 0–2 components). Results from these sensitivity analyses are presented in the Supplementary Materials (Table S1). All data analyses were performed using STATA version 17.

## Results

**Figure 2.**
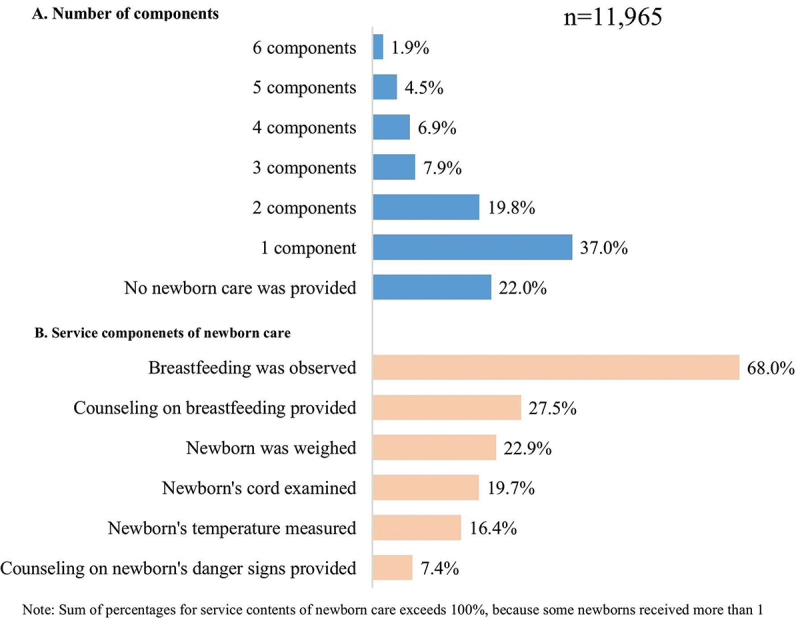
Number of components (A) and service components (B) of early newborn postnatal care (PNC) services.

Figure 2 illustrates the distribution of early newborn PNC services among 11,965 ever-married women. Overall, 41.0% (95% CI 39.6%–42.5%) of infants received ≥2 components of early newborn PNC services. However, only 1.9% of newborns received all six recommended components, while 76.1% received between one and five services, and 22.0% did not receive any of the six components. Regarding specific service components, the most commonly reported services were observation of breastfeeding (68.0%), breastfeeding counseling (27.5%), and weighing of the newborn (22.9%). In contrast, umbilical cord examination, temperature measurement, and counseling on newborn danger signs were reported by 19.7%, 16.4%, and 7.4% of respondents, respectively.

Table 1 presents the characteristics of mothers and infants according to whether the newborn received ≥2 components. Most women (60.6%) were aged 15–29 years. Significant differences were observed between the ≥2 components and 0–1 component groups across several sociodemographic and clinical variables. Women in the ≥2-component group were more likely to have formal education, belong to households with an educated head, reside in urban areas, and be in higher wealth quintiles. Additionally, they were more likely to have utilized ANC, delivered in a health facility, undergone cesarean section, and had access to media.

**Table 1.** Baseline characteristics of the mother–infant dyads.

Characteristics	Total (weighted %) *n* = 11,965	Received ≥ 2 newborn PNC components		*p*-Value
		Yes (weighted %) *n* = 5,068 (41.0)	No (weighted %) *n* = 6,897 (59.0)	
Women’s age (years)				
15–29	60.6	62.8	59.0	0.005
30–39	32.3	31.0	33.3	
40–49	7.1	6.2	7.7	
Women’s education				
No formal education	75.9	71.0	79.2	<0.001
Attended formal education	24.1	29.0	20.8	
Household head education				
No formal education	62.1	54.6	67.3	<0.001
Attended formal education	37.9	45.4	32.7	
Residential area				
Urban	23.2	29.2	19.0	<0.001
Rural	76.8	70.8	81.0	
Place of delivery				
Home	33.6	18.1	44.4	<0.001
Public health facility	56.0	69.6	46.5	
Private health facility	10.4	12.3	9.1	
Antenatal care (ANC) use				
No visits	22.1	14.4	27.5	<0.001
1–3 visits	44.5	43.3	45.3	
≥4 visits	33.4	42.3	27.3	
Sex of infant				
Male	51.6	51.9	51.3	0.633
Female	48.4	48.1	48.7	
Birth order				
1^st^ child	16.0	19.0	14.0	<0.001
2nd or higher child	84.0	81.0	86.0	
Cesarean section				
No	94.4	91.4	96.5	<0.001
Yes	5.6	8.6	3.5	
Small-sized baby				
No	72.8	75.2	71.1	<0.001
Yes	27.2	24.8	28.9	
Women’s access to media				
No	79.5	73.8	83.4	<0.001
Yes	20.5	26.2	16.6	
Wealth status				
Lowest quintile	20.8	14.0	25.5	<0.001
Second	20.9	18.4	22.6	
Third	20.4	19.8	20.8	
Fourth	19.5	21.8	18.0	
Highest quintile	18.5	26.0	13.2	

Abbreviations: PNC: Postnatal care.

Table 2 presents the adjusted odds of receiving ≥2 components of early newborn PNC across various explanatory variables. Infants from households with an educated head had significantly higher odds of receiving ≥2 components of PNC services (AOR: 1.25, 95% CI: 1.10–1.42). Compared to home births, the odds of receiving ≥2 components of PNC was substantially higher for infants born at private (AOR: 2.13, 95% CI: 1.70–2.68) and public (AOR: 2.71, 95% CI: 2.38–3.08) health facilities. Similarly, infants whose mothers had 1–3 (AOR: 1.25, 95% CI: 1.08–1.45) and ≥4 (AOR: 1.68, 95% CI: 1.42–1.99) ANC visits were more likely to receive ≥2 components of PNC services. Delivery by cesarean section was also associated with higher odds of receiving ≥2 components of PNC (AOR: 1.46, 95% CI: 1.13–1.89). Moreover, a positive association was found across increasing wealth quintiles, with infants in the second (AOR: 1.24, 95% CI: 1.02–1.50), third (AOR: 1.21, 95% CI: 1.00–1.46), fourth (AOR: 1.33, 95% CI: 1.07–1.65) and fifth (AOR: 1.77, 95% CI: 1.35–2.33) quintiles being more likely to receive ≥2 components of PNC compared to those in the poorest quintile. In contrast, infants who were perceived as small at birth had lower odds (AOR: 0.86, 95% CI: 0.75–0.99) of receiving ≥2 components of PNC.

**Table 2.** Likelihood of receiving ≥2 components of early newborn postnatal care (PNC) services.

Characteristics	COR (95%CI)	*p*-Value	AOR (95%CI)	*p*-Value
Women’s age (years)				
15–29	Reference		Reference	
30–39	0.88 (0.78–0.98)	0.026	0.95 (0.83–1.08)	0.438
40–49	0.76 (0.63–0.92)	0.005	0.89 (0.72–1.09)	0.255
Women’s education				
No formal education	Reference		Reference	
Attended formal education	1.56 (1.37–1.77)	<0.001	0.90 (0.78–1.05)	0.183
Household head education				
No formal education	Reference		Reference	
Attended formal education	1.71 (1.52–1.92)	<0.001	**1.25 (1.10–1.42)**	**0.001**
Residential area				
Urban	Reference		Reference	
Rural	0.57 (0.49–0.66)	<0.001	1.05 (0.88–1.27)	0.574
Place of delivery				
Home	Reference		Reference	
Public health facility	3.67 (3.25–4.15)	<0.001	**2.71 (2.38–3.08)**	**<0.001**
Private health facility	3.30 (2.65–4.12)	<0.001	**2.13 (1.70–2.68)**	**<0.001**
Antenatal care (ANC) use				
No visits	Reference		Reference	
1–3 visits	1.83 (1.59–2.10)	<0.001	**1.25 (1.08–1.45)**	**0.003**
≥4 visits	2.96 (2.52–3.49)	<0.001	**1.68 (1.42–1.99)**	**<0.001**
Sex of infant				
Male	Reference		–	–
Female	0.98 (0.88–1.08)	0.634	–	–
Birth order				
1^st^ child	Reference		Reference	
2nd or higher child	0.69 (0.58–0.82)	<0.001	0.84 (0.69–1.02)	0.079
Cesarean section				
No	Reference		Reference	
Yes	2.57 (1.99–3.33)	<0.001	**1.46 (1.13–1.89)**	**0.004**
Small-sized baby				
No	Reference		Reference	
Yes	0.81 (0.71–0.92)	0.001	**0.86 (0.75–0.99)**	**0.032**
Women’s access to media				
No	Reference		Reference	
Yes	1.79 (1.51–2.13)	<0.001	1.08 (0.89–1.30)	0.460
Wealth status				
Lowest quintile	Reference		Reference	
Second	1.49 (1.23–1.81)	<0.001	**1.24 (1.02–1.50)**	**0.028**
Third	1.75 (1.45–2.11)	<0.001	**1.21 (1.00–1.46)**	**0.049**
Fourth	2.22 (1.82–2.71)	<0.001	**1.33 (1.07–1.65)**	**0.009**
Highest quintile	3.62 (2.90–4.50)	<0.001	**1.77 (1.35–2.33)**	**<0.001**

Abbreviations: COR: Crude odds ratio, AOR: Adjusted odds ratio. Statistically significant values are shown in bold.

Sensitivity analyses using alternative thresholds produced broadly similar patterns for the principal associations (Table S1).

## Discussion

This study examined the receipt of early newborn PNC services and its associated factors in Afghanistan, using nationally representative data. While 41.0% of infants were found to have received at least two components of early newborn PNC services, only 1.9% received all six components of the early newborn PNC services. Key factors positively associated with receiving ≥2 components of early newborn PNC included higher education of the household head, greater ANC utilization, facility-based delivery, cesarean section, and higher household wealth. Conversely, newborns perceived as small at birth were less likely to receive ≥2 components of early newborn PNC services.

The finding that 41.0% of infants in Afghanistan received at least two components of early newborn PNC services aligns with similar estimates from other LMICs, such as Ethiopia (39.6%) [21] and Uganda (50%) [23]. To date, no nationally representative studies in Afghanistan have specifically assessed the receipt of early newborn PNC services. Earlier studies in Afghanistan have mainly focused on PNC services for women [27,34,35] or individual components of early newborn care, such as skin-to-skin contact, breastfeeding initiation, and cord care [19,28,29]. In this study, while relatively higher proportions of mothers reported receiving services such as breastfeeding counseling, observation during breastfeeding, newborn weighing, and cord examination, each reported by approximately 20% or more of mothers, whereas temperature measurement and counseling on neonatal danger signs were reported by fewer than 20%. These differences should be interpreted cautiously because some provider-performed procedures, such as temperature measurement or cord examination, may occur without being clearly communicated to the mother and may, therefore, be less easily noticed or recalled. In contrast, practices that directly involve both the mother and newborn, such as breastfeeding counselling or observation of breastfeeding, may be easier to remember. However, these service-specific disparities are consistent with existing literature from LMICs, which highlights the variability in the content and quality of PNC received [7,16,18,19,25,36,37]. Given the well-established evidence that comprehensive early newborn PNC significantly reduces neonatal morbidity and mortality [16,18,38,39], these gaps underscore the urgent need for targeted interventions and policy reforms in Afghanistan to improve early newborn PNC services.

We found that infants from households with educated heads had about 1.2-fold higher odds of receiving at least two components of early newborn PNC services than those from households where the head had no formal education. This association aligns with findings from studies conducted in other LMICs, such as East Africa [9], Ethiopia [21], and Bangladesh [40]. Previous studies in Afghanistan have also supported the crucial role of household head education in the optimal utilization of maternal and child healthcare services [28,34,41,42]. Educated heads of households are more likely to possess better health literacy, enabling informed decision-making about seeking timely and appropriate care for newborns. Additionally, they may have greater autonomy, resources, and exposure to media or health promotion messages that support the uptake of PNC services [43,44]. These findings highlight the importance of considering the educational status of household decision-makers in the design of interventions to improve early newborn PNC utilization.

Maternal education was associated with the receipt of at least two components of early newborn PNC services in the unadjusted analysis but did not remain significant after adjustment. Formal education was uncommon among women in the study, reflecting the longstanding barriers Afghan women have faced in accessing education [14]. Maternal education may also influence newborn care indirectly through health literacy, household resources, decision-making power, ANC attendance, and facility delivery [45,46]. Because several of these related factors were included in the adjusted model, they may have accounted for part of the association. This finding should, therefore, not be interpreted as evidence that women’s education is less important. Continued restrictions on girls’ and women’s education may worsen maternal and newborn health in the future by limiting the knowledge, autonomy, and healthcare access of future mothers, while also reducing the number of educated female health professionals available to provide culturally acceptable maternal and newborn services [14,46].

We found that infants whose mothers used ANC services had higher odds of receiving at least two components of early newborn PNC services. This finding aligns with a systematic review from East Africa that linked ANC attendance to higher uptake of early PNC components [26], and with other studies in LMICs underscoring ANC as a critical platform for preparing mothers and families for postnatal care [26,32,47]. Additionally, previous studies in Afghanistan have demonstrated that ANC utilization enhances mothers’ awareness of and access to essential newborn services, including immunizations and growth monitoring [19,29,45]. However, adequate ANC utilization remains suboptimal in Afghanistan [42,48,49], and the country remains far from achieving the WHO recommendation of eight ANC contacts. Reductions in donor funding, shortages of female health workers, and the closure or reduced functioning of health facilities may further limit the availability and accessibility of ANC services, particularly in rural and underserved areas [46,50]. Moreover, restrictions on women’s travel without a male family member may reduce access to routine ANC, especially for women living far from health facilities or in poor households, where limited resources and accompaniment may be prioritized for emergency care rather than preventive services [46,51]. These restrictions may also limit women’s participation in group ANC and reduce the reach of community-based ANC outreach. To bridge these gaps, strategies should prioritize improving both the coverage and quality of ANC by strengthening Basic Package of Health Services (BPHS), expanding community and mobile outreach, providing culturally appropriate group ANC, and integrating PNC counselling into routine antenatal sessions, as a means to reduce inequities and improve early newborn PNC uptake.

We found that infants born in public and private health facilities had 2.7 and 2.1-fold higher odds, respectively, of receiving at least two components of early newborn PNC services compared to those born at home. This finding is consistent with previous studies conducted in Ethiopia [21,52], Bangladesh [53], and India [54]. Newborns delivered in health facilities may receive essential newborn care, including immediate drying of the baby, cord care, timely initiation of breastfeeding, and skin-to-skin contact after birth [28,29,36]. Healthcare workers may also provide mothers with important information about PNC services for both themselves and their newborns [1]. However, this association should be interpreted cautiously because several components included in the outcome, such as weighing, temperature measurement, cord examination, and breastfeeding observation, are more readily provided during routine facility-based care before discharge. Thus, the place of delivery and receipt of these components may not be fully independent, and the observed association may partly reflect greater contact with skilled providers and the construction of the outcome rather than an independent causal effect of facility delivery. Although the institutional delivery rate in Afghanistan remains low, approximately 50% [34], expanding access to facility-based delivery should be accompanied by measures to ensure consistent provision of recommended newborn PNC components before discharge and timely home- or community-based follow-up for infants born outside health facilities.

Cesarean delivery also remained associated with higher odds of receiving at least two components of early newborn PNC, a relationship noted in other settings such as Ethiopia [21]. While facility-based surgical births inherently involve prolonged contact with skilled providers, who can deliver and document essential newborn interventions [1,55], studies in Afghanistan have paradoxically identified suboptimal newborn care following cesarean section [28,29]. This discrepancy may reflect gaps in postoperative PNC protocols, staff training, or resource constraints in operating theatres and recovery wards. To address this, policymakers should develop and enforce standardized PNC checklists and protocols specifically tailored for post-cesarean settings, ensure dedicated staff training on newborn care in surgical units, and monitor compliance through routine facility audits and supportive supervision.

The result showed that infants perceived by their mothers as small at birth had lower odds of receiving at least two components of early newborn PNC services. This finding should be interpreted with caution. Although infants perceived as small at birth are at increased risk of adverse neonatal outcomes [9,38,54] and would be expected to benefit from comprehensive PNC services, the MICS dataset does not include information on gestational age, prematurity, neonatal complications, and resuscitation at birth, hospitalization, referral, or specialized neonatal care. Consequently, some small-sized infants may have followed clinical pathways that differ from routine PNC services, and the six PNC components examined in this study may not fully capture the care they received. Therefore, the observed association may partly reflect differences in clinical management rather than inequitable access to PNC services. Nevertheless, previous evidence from Afghanistan has documented low coverage of several essential newborn care practices such as skin-to-skin contact among vulnerable newborns, including small and preterm infants [19,28,29], suggesting that improving the quality and continuity of care for these high-risk newborns remains an important priority.

In this study, women’s access to media was significantly associated with the receipt of at least two components of early newborn PNC in the unadjusted analysis but was no longer significant in the adjusted model. One possible explanation is that women with access to media were also more likely to belong to wealthier households and have better access to education and health services [49]. Once household wealth and other related factors were included in the multivariable model, media access did not show an independent association with the outcome. Therefore, the initial association may have reflected the socioeconomic advantages of women with media access rather than the effect of media exposure alone.

Lastly, our analysis demonstrated a clear wealth gradient in early newborn PNC uptake: infants from the second through fifth quintiles were progressively more likely to receive at least two components of PNC services compared to those in the poorest quintile. This inequity is not unique to newborn PNC; previous studies in Afghanistan have also documented substantial wealth-related gaps in ANC utilization, skilled birth attendance, maternal PNC, and other maternal and child health services [21,34,56,57]. Households with greater financial resources face fewer barriers to accessing facility-based services, such as transportation costs, informal fees, and opportunity costs that can deter PNC visits. To bridge this gap, policies should aim to reduce out-of-pocket expenditures, for example, through voucher schemes or conditional cash transfers for postnatal visits, alongside community-based initiatives targeting the poorest families. Additionally, expanding free or subsidized newborn care packages and strengthening outreach by community health workers can help ensure equitable access to early PNC regardless of household income.

## Limitations

Despite its strengths, this study has some limitations. First, all data in MICS were self-reported and may, therefore, be subject to information and recall biases. Recall may have been particularly difficult for technical procedures such as temperature measurement, cord examination, and counseling on newborn danger signs. Second, the measurement of early newborn PNC may have differed according to place of delivery. Approximately 33.6% of births occurred at home, and some home-born newborns may subsequently have been assessed by community health workers, traditional birth attendants, or health-facility providers within the first 48 hours. Moreover, several measured components, including newborn weighing, temperature measurement, cord examination, and breastfeeding observation, are more readily provided during routine facility-based care. Consequently, the component-based outcome may partly reflect access to skilled providers and health-facility infrastructure rather than differences in PNC content alone. Place of delivery and receipt of the measured components may, therefore, not be fully independent, and the strong association observed for facility delivery should not be interpreted as an independent causal effect. Third, our evaluation of the factors associated with receipt of early newborn PNC components was limited to variables available in the MICS dataset. Other potentially important factors, such as newborn health, distance to a healthcare facility, health expenditures, and traditional and cultural beliefs regarding newborn PNC services, were not assessed in the present study. Therefore, future studies should consider these critical factors in their analyses. Fourth, the MICS 2022–2023 questionnaire did not capture the full range of early newborn PNC services recommended by the 2022 WHO guidelines; most notably, congenital disease screening, potentially underestimating service comprehensiveness. Finally, the cross-sectional design precludes establishing temporality or causality between receipt of early newborn PNC components and the associated factors. Nevertheless, by identifying critical gaps in both service content and equity of access, these findings can guide the development of targeted policies and interventions aimed at strengthening the quality and reach of early newborn PNC services in Afghanistan.

## Conclusion

The uptake of early newborn PNC services in Afghanistan remains low, with four out of ten infants receiving at least two PNC components within the first 48 hours after birth. Key factors associated with the receipt of early newborn PNC services included household head education, ANC utilization, facility-based delivery (including cesarean section), perceived infant size at birth, and household wealth. These findings highlight priority areas for policy and programmatic interventions, such as enhancing health literacy among household decision‑makers, expanding and improving the quality of ANC and facility delivery services, and implementing targeted support for infants who are perceived as small at birth. Future research should also explore the roles of cultural norms and religious beliefs in shaping early newborn PNC utilization to further tailor interventions for the Afghan context.

## Supplementary Material

Supplementary Materials.docx

## Data Availability

The MICS 2022–23 dataset is publicly available on UNICEF’s official website through the following link: https://mics.unicef.org/surveys?display=card&keys=Afghanistan.
