# Supplementary material for: Coverage and factors associated with receipt of early newborn postnatal care components in Afghanistan: findings from the 2022–2023 multiple indicator cluster survey
Source: Glob Health Action. 2026 Aug 3;19(1):2710553. doi: 10.1080/16549716.2026.2710553 (PMC13435275; doi:10.1080/16549716.2026.2710553)
Supplement: Supplementary file 1 [file ZGHA_A_2710553_SM9121.docx]

| Table S1. Sensitivity analysis on the likelihood of receipt of ≥4 components and ≥3 components of newborn PNC | | | | | |
| --- | --- | --- | --- | --- | --- |
|  |  | ≥4 components vs.  0-3 components | p-value | ≥3 components vs.  0-2 components | p-value |
|  |  | Adjusted odds ratio  (95% CI)  n= 11965 |  | Adjusted odds ratio  (95% CI)  n= 11965 |  |
| Women age | 15-29 years (ref) | 1 |  | 1 |  |
|  | 30-39 years | 1.04 (0.87-1.26) | 0.646 | 1.00 (0.85-1.18) | 0.995 |
|  | 40-49 years | 1.15 (0.86-1.55) | 0.346 | 1.01 (0.79-1.30) | 0.937 |
| Women | No formal education (ref) | 1 |  | 1 |  |
| education | Attended formal education | 0.94 (0.75-1.17) | 0.560 | 0.89 (0.74-1.07) | 0.208 |
| Head of household  education | No formal education (ref) | 1 |  | 1 |  |
|  | Attended formal education | **1.29 (1.07-1.55)** | **0.008** | **1.28 (1.11-1.49)** | **0.001** |
| Wealth index | 1^st^ quintile (ref) | 1 |  | 1 |  |
|  | 2^nd^ quintile | 0.99 (0.73-1.34) | 0.962 | 1.08 (0.86-1.36) | 0.524 |
|  | 3^rd^ quintile | 1.12 (0.80-1.58) | 0.497 | 1.16 (0.89-1.50) | 0.282 |
|  | 4^th^ quintile | 1.40 (0.98-2.00) | 0.061 | **1.32 (1.01-1.73)** | **0.043** |
|  | 5^th^ quintile | **2.15 (1.42-3.26)** | **<0.001** | **1.94 (1.39-2.69)** | **<0.001** |
| Residential area | Urban (ref) | 1 |  | 1 |  |
|  | Rural | 1.17 (0.89-1.54) | 0.259 | 1.13 (0.91-1.40) | 0.279 |
| Place of delivery | Home (ref) | 1 |  | 1 |  |
|  | Government facilities | **4.32 (3.35-5.58)** | **<0.001** | **3.10 (2.57-3.75)** | **<0.001** |
|  | Private facilities | **4.15 (2.98-5.76)** | **<0.001** | **2.55 (1.92-3.37)** | **<0.001** |
| ANC use | No ANC visit (ref) | 1 |  | 1 |  |
|  | 1-3 ANC visits | 1.12 (0.87-1.43) | 0.374 | 1.09 (0.89-1.33) | 0.425 |
|  | ≥4 ANC visits | **1.46 (1.12-1.91)** | **0.005** | **1.57 (1.27-1.94)** | **<0.001** |
| Access to media | No (ref) | 1 |  | 1 |  |
|  | Yes | 1.15 (0.92-1.44) | 0.229 | 1.12 (0.93-1.35) | 0.236 |
| Number of children | One child (ref) | 1 |  | 1 |  |
| women gave birth | ≥ 2 children | 0.91 (0.71-1.16) | 0.446 | 0.97 (0.79-1.19) | 0.776 |
| Small size baby | No (ref) | 1 |  | 1 |  |
|  | Yes | 0.80 (0.63-1.01) | 0.064 | 0.86 (0.72-1.01) | 0.072 |
| Caesarean section | No (ref) | 1 |  | 1 |  |
|  | Yes | **1.49 (1.10-2.00)** | **0.009** | **1.47 (1.13-1.92)** | **0.004** |

Weighted % (95% CI) of ≥4 and ≥3 components of newborn PNC, were 13.3 (12.3-14.4) and 21.2 (20.0-22.5), respectively.
